# Targeted photodestruction of human colon cancer cells using charged Dougherty chlorin*_e6_*immunoconjugates

**DOI:** 10.1054/bjoc.1999.0877

**Published:** 1999-12-08

**Authors:** M Del Governatore, MR Hamblin, EE Piccinini, G Ugolini, T Hasan

**Affiliations:** Wellman Laboratories of Photomedicine, Department of Dermatology, Massachusetts General Hospital, Harvard Medical School, Boston, MA 02114, USA; Department of Chirurgia III, Ospedale Policlinico S. Orsola Via Massarenti 9, Bologna, 40100, Italy

**Keywords:** photodynamic therapy, photoimmunotherapy, photosensitizer, polylysine, laser

## Abstract

The goal of this study was to develop a strategy for the selective destruction of colorectal cancer cells. Towards this end, photoimmunoconjugates were prepared between the anti-colon cancer monoclonal antibody 17.1A and the photosensitizer (PS) chlorin*_e6_*(c*_e6_*). Polylysine linkers bearing several c*_e6_*molecules were covalently attached in a site-specific manner to partially reduced IgG molecules, which allowed photoimmunoconjugates to bear either cationic or anionic charges. The conjugates retained immunoreactivity as shown by enzyme-linked immunosorbent assays and by competition studies with native antibody. The overall charge on the photoimmunoconjugate was an important determinant of PS delivery. The cationic photoimmunoconjugate delivered 4 times more c*_e6_*to the cells than the anionic photoimmunoconjugate, and both 17.1A conjugates showed, in comparison to non-specific rabbit IgG conjugates, selectivity for antigen-positive target cells. Illumination with only 3 J cm^−2^of 666 nm light reduced the number of colony forming cells by more than 90% for the cationic 17.1A conjugate and by 73% for the anionic 17.1A conjugate after incubation with 1 μM c*_e6_*equivalent of the respective conjugates. By contrast, 1 μM free c*_e6_*gave only a 35% reduction in colonies. These data suggest photoimmunoconjugates may have applications in photoimmunotherapy where destruction of colorectal cancer cells is required. © 2000 Cancer Research Campaign


*British Journal of Cancer* (2000) **82**(1), 56–64
© 2000 Cancer Research Campaign
Article no. bjoc.1999.0877

### Targeted photodestruction of human colon cancer cells using charged 17.1A chlorin$_{e6}$ immunoconjugates

**M Del Governatore[1,2], MR Hamblin[1], EE Piccinini[2], G Ugolini[2] and T Hasan[1]**

[1]Wellman Laboratories of Photomedicine, Department of Dermatology, Massachusetts General Hospital, Harvard Medical School, Boston, MA 02114, USA; [2]Department of Chirurgia III, Ospedale Policlinico S. Orsola, Via Massarenti 9, Bologna 40100 Italy

**Summary** The goal of this study was to develop a strategy for the selective destruction of colorectal cancer cells. Towards this end, photoimmunoconjugates were prepared between the anti-colon cancer monoclonal antibody 17.1A and the photosensitizer (PS) chlorin$_{e6}$ (c$_{e6}$). Polylysine linkers bearing several c$_{e6}$ molecules were covalently attached in a site-specific manner to partially reduced IgG molecules, which allowed photoimmunoconjugates to bear either cationic or anionic charges. The conjugates retained immunoreactivity as shown by enzyme-linked immunosorbent assays and by competition studies with native antibody. The overall charge on the photoimmunoconjugate was an important determinant of PS delivery. The cationic photoimmunoconjugate delivered 4 times more c$_{e6}$ to the cells than the anionic photoimmunoconjugate, and both 17.1A conjugates showed, in comparison to non-specific rabbit IgG conjugates, selectivity for antigen-positive target cells. Illumination with only 3 J cm$^{-2}$ of 666 nm light reduced the number of colony forming cells by more than 90% for the cationic 17.1A conjugate and by 73% for the anionic 17.1A conjugate after incubation with 1 μM c$_{e6}$ equivalent of the respective conjugates. By contrast, 1 μM free c$_{e6}$ gave only a 35% reduction in colonies. These data suggest photoimmunoconjugates may have applications in photoimmunotherapy where destruction of colorectal cancer cells is required. © 2000 Cancer Research Campaign

**Keywords:** photodynamic therapy; photoimmunotherapy; photosensitizer; polylysine; laser

Photodynamic therapy (PDT) is an experimental approach for cancer treatment in which the local or systemic delivery of a photosensitizer (PS) is followed by tissue illumination with light of an appropriate wavelength (usually red light delivered by a laser) (Hasan and Parrish, 1996; Dougherty et al, 1998). Conventional PS may have some selective accumulation in tumours (Henderson and Dougherty, 1992; Hamblin and Newman, 1994), but increased tumour targeting may be obtained by the use of macromolecular carriers which form complexes or covalent conjugates with PS (Hasan, 1992). The targeting capability of these carriers may rely on physical properties of the carrier (liposomes and microspheres (Speiser, 1991)), molecular properties (molecular weight and charge (Kornguth et al, 1989)) or the specific recognition of molecules associated with tumours (monoclonal antibodies (mAbs), lipoproteins and growth factor receptor ligands). They have been used to deliver cytotoxic drugs (Johnson et al, 1995), radioisotopes (Buchsbaum et al, 1993*a*), protein toxins (Houston, 1993) and PS to tumours, but the latter has the distinct advantage of not being toxic until illuminated with activating light, thus reducing toxicity due to non-specific uptake of the conjugate which can be a problem with conjugates formed from toxins and radioisotopes. Conjugates between mAbs and PS have shown promise both in vitro (Pogrebniak et al, 1993; Vrouenraets et al, 1999) and in experimental animal models of cancer (Mew et al, 1983; Goff et al, 1996), but have received only minimal clinical testing (Schmidt et al, 1992). The charge borne by the immunoconjugate may markedly influence the pharmacokinetics and biodistribution (Slinkin et al, 1993), and manipulation of the overall charge may increase the therapeutic ratio.

*Received 20 April 1999*
*Revised 13 July 1999*
*Accepted 16 July 1999*

*Correspondence to:* T Hasan

Colorectal cancer is in need of novel and effective therapies, and certain aspects of it may be appropriate for treatment by PDT. The ability to selectively target colorectal cancer cells may have applications to the tumour bed after surgical resection of the primary tumour (Barr et al, 1990), to disseminated intraperitoneal carcinomatosis (Veenhuizen et al, 1996), and to the liver metastases (Van Hillegersberg et al, 1992*a*) which are a frequent cause of death (Benotti and Steele, 1992). Because of the sensitive tissues in the peritoneal cavity (Veenhuizen et al, 1997), and the high accumulation of conventional PS in normal liver (Van Hillegersberg et al, 1992*b*), it is attractive to explore the use of photoimmunoconjugates (PICs) constructed from mAbs targeted against colorectal cancer antigens, to increase the selectivity of the PS for tumour over normal tissue. The epithelial membrane antigen (a homophilic cell–cell adhesion molecule) which is recognized by several mAbs (including 17.1A) is overexpressed on many cancers of the gastrointestinal tract (Litvinov et al, 1994). The murine monoclonal IgG 17.1A has been used clinically to treat human colorectal cancer both in an unconjugated form to induce antibody-dependent cellular cytotoxicity (Riethmuller et al, 1994) and as radioimmunoconjugates to target radioisotopes to residual tumour (Buchsbaum et al, 1993*b*). As part of our on-going effort to optimize intraperitoneal photoimmunotherapy (PIT) (Goff et al, 1991, 1994, 1996) we have recently described the preparation of charged PICs between the F(ab′)$_2$ fragment of anti-ovarian cancer Mab OC125 and the PS chlorin$_{e6}$ (c$_{e6}$) (Hamblin et al, 1996), and have investigated their biodistribution in vivo (Duska et al, 1997). Toward the long-term goal of applying PIT to intraperitoneal and hepatic metastases of colorectal cancer, we report here our initial studies on the preparation of charged PICs with the intact mAb 17.1A and their in vitro interactions with a human colorectal cancer cell line recognized by the mAb (HT29) and a cell line which showed no binding to the mAb (OVCAR-5).

### MATERIALS AND METHODS

### Cell line and monoclonal antibody

Two tumour cells lines were employed. HT29 cells derived from a human colorectal adenocarcinoma were a generous gift from Dr K Tanabe (Massachusetts General Hospital, Boston, MA, USA). NIH:OVCAR-5 human ovarian cancer cells were purchased from Dr T Hamilton (Fox Chase Cancer Center, Philadelphia, PA, USA). The growth medium for HT29 was DMEM/F12 (50/50 mixture) and that for OVCAR-5 was RPMI-1640. Both media contained 15 mM HEPES and L-glutamine and were supplemented with 10% heat-inactivated fetal calf serum (FCS) (Whittaker Bioproduct, Walkersville, MD, USA), 100 U ml$^{-1}$ penicillin and 100 μg ml$^{-1}$ streptomycin, and maintained in an incubator at 37°C in an atmosphere of 5% carbon dioxide. 17.1A murine mAb was a kind gift from Centacor (Malvern, PA, USA). Rabbit IgG and mouse IgG was obtained from Sigma (St Louis, MO, USA).

### Conjugation procedure

The procedure has been described in detail elsewhere (Hamblin et al, 1996). Briefly poly-L-lysine (average MW 25 000) was treated in DMSO with the N-hydroxysuccinimide ester of $c_{e6}$ to give pl-$c_{e6}$. This was then reacted with pyridyldithiopropionic acid N-hydroxysuccinimide ester (SPDP) to form the functionalized derivative pl-$c_{e6}$-SPDP. This was then split into two parts and one part was treated with an excess of succinic anhydride to give the negatively charged functionalized pl-$c_{e6}$-succ-SPDP. 17.1A mAb was reduced for 1 h with 5 mM mercaptoethylamine hydrochloride, dialysed (1 mM EDTA) and then reacted with either pl-$c_{e6}$-SPDP or pl-$c_{e6}$-succ-SPDP to form the cationic and anionic PICs respectively. The procedure was repeated with rabbit IgG. The conjugates were purified by chromatography on Sephadex G200 columns, and characterized by absorption and fluorescence spectrophotometry, and polyacrylamide gel electrophoresis. The structures of the PICs are shown in Figure 1.

### Enzyme-linked immunosorbent assay

Cells were grown to 100% confluence in 96-well plates for 24 h with medium containing FCS, than washed 3 times with PBS and fixed with 0.25% glutaraldehyde. After 1 h cells were washed with PBS and plates blocked with PBS containing 5% FCS for 1 h, then 0.1 ml PBS containing the appropriate dilution of PIC or mAb was added to each well. After 2 h incubation at room temperature in the dark, wells were washed 3 times with PBS containing 0.05% Tween 20 and had added 0.1 ml of horseradish peroxidase conjugated-F(ab′)$_2$ fragment rabbit anti mouse IgG, IgA, IgM (h+l) (Zymed Laboratories, South San Francisco, CA, USA) diluted 1:200 in PBS containing 0.5 mg ml$^{-1}$ bovine serum albumin (BSA) and 0.05% Tween-20. Cells were incubated for 2 h at room temperature in the dark then washed 3 times with PBS containing 0.05% Tween-20 and added 0.1 ml of 0.4 mg ml$^{-1}$ of freshly prepared o-phenylenediamine dissolved in 0.05 M sodium citrate, 0.15 M sodium phosphate, pH 6, containing 32% vol/vol of 30% hydrogen peroxide and incubated for a further hour at room temperature in the dark. Absorbance was read at 492 nm with an enzyme-linked immunosorbent assay (ELISA) reader (500 EIA, Bio-Rad Laboratories, Hercules, CA, USA).

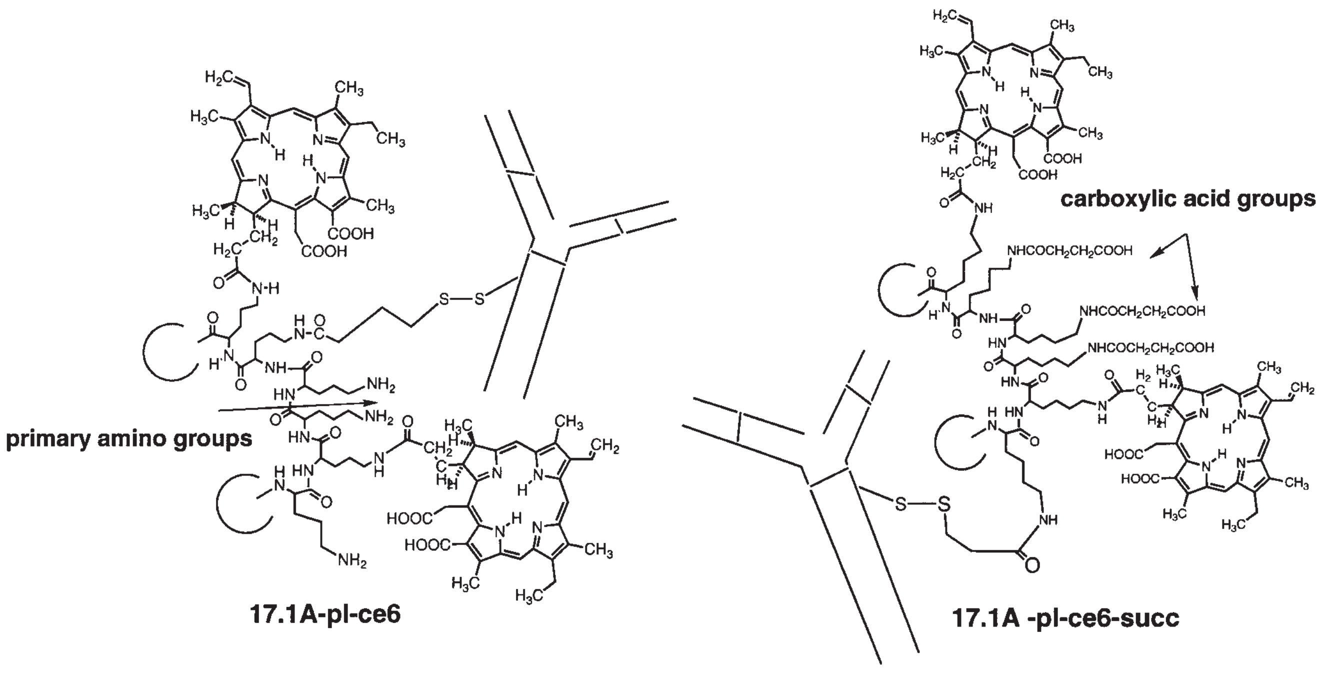

**Figure 1** Structures of the PICs. 17.1A-pl-$c_{e6}$ has primary amino groups which give it a polycationic charge, while 17.1A-pl-$c_{e6}$-succ has carboxylic groups which give it polyanionic charge

© 2000 Cancer Research Campaign

### Two-colour direct/indirect immunofluorescence

Approximately $3 \times 10^5$ cells were plated in 35-mm tissue culture dishes, for 24 h with 2 ml of medium, containing coverslips that were previously washed with 95% ethanol and flamed. After 24 h of incubation in 2 ml of medium, cells were washed with PBS (2×) and fixed with 2% formaldehyde at room temperature for 5 min. Cells were washed with PBS/1% BSA (3×), and incubated for 1 h at room temperature with the mAb or conjugate, the amount of mAb in both cases was 2 μg $ml^{-1}$. After this time they were gently washed with PBS/1% BSA (3×) and incubated for 1 h with or without fluorescein isothiocyanate-conjugated goat anti-mouse IgG (FITC-GAM, Sigma) diluted 1:128 with PBS/1% BSA. Cells were washed with PBS/1% BSA (3×) and mounted on glass microscope slides using GelMount (Biomeda Corp., Foster City, CA, USA). An epi-illumination microscope (Model WL, Zeiss, Oberkochen, Germany) equipped with a CCD camera (TM 745, Pulnix, Sunnyvale, CA, USA), image intensifier (M942, Litton Electron Devices, Tempe, AZ, USA), video monitor and computer was used to capture digital images. Two different combinations of filters were used. The first set used an excitation bandpass filter at 450–490 nm and emission 514–530 nm bandpass filter designed for visualizing fluorescein fluorescence. The second set used an excitation bandpass filter at 402–447 nm and emission 580 nm longpass filter designed for visualizing $c_{e6}$ fluorescence. The images obtained with the $c_{e6}$ filter combination viewing cells incubated with unconjugated 17.1A and FITC-GAM were totally negative, while those obtained with cells incubated with a $c_{e6}$ conjugate without FITC-GAM and the fluorescein filter set gave a faint image.

### Cellular uptake

Twelve-well plates containing 90% confluent cells had 1 ml medium containing 10% FCS and conjugates added. After the completion of the incubation time cells were washed with PBS (3×) and incubated with trypsin (0.25%)/EDTA (0.02%) (1 ml) at 37°C for 20 min. The suspension was centrifuged and the resulting cell pellet dissolved for 48 h in 1.5 ml of 0.1 M sodium hydroxide (NaOH); 1% sodium dodecyl sulphate (SDS) to give a homogeneous solution. Fluorescence was measured with a fluorometer (Fluorolog 2, Spex Industries, Edison, NJ, USA) (excitation at 400 nm, emission scanned from 580 to 720 nm). The trypsin supernatant was checked for the presence of fluorescence, which was always less than 10% of the cell extract. The cell digest was then assayed for the amount of cell protein by a modified Lowry procedure (Larson et al, 1986). Quantitation of $c_{e6}$ concentration in the cell extracts was obtained by comparing the fluorescence of standard solutions of the same conjugate of known concentrations in 0.1 M NaOH 1% SDS. The fluorescence of the cell extracts was always within the linear part of the standard curve. Uptake experiments at 4°C were carried out by adding pre-cooled medium containing conjugates to the wells in 12-well plates, which were then wrapped in aluminium foil and incubated in crushed ice for 6 h.

### Competition

Studies were conducted in which the cells were preincubated with a saturating concentration of unmodified 17.1A, to see if the binding of the PICs was blocked. Since 1 ml of 1 μM $c_{e6}$ equivalent PIC contains approximately 7.5 μg mAb, a fivefold excess of competing 17.1A or rabbit IgG (37.5 μg) was employed. The conjugate was added at a concentration of 1 μM $c_{e6}$ equivalent and incubated for 2 h. The uncompeted uptake was compared to the uptake found by the addition of 17.1A or rabbit IgG (37.5 μg $ml^{-1}$) for 1 h, followed by washing and addition of PIC (1 μM $c_{e6}$ equivalent) for 1 h.

### Phototoxicity

Phototoxicity was measured by a colony forming assay. In P35 dishes cells were grown to 70% confluence in 2 ml medium containing 10% FBS. Cells were washed with PBS and 1 μM $c_{e6}$ equivalent of PICs and PS in serum containing medium was added. After 6 h incubation at 37°C, the medium was removed, cells were washed twice with PBS, and 2 ml of fresh serum containing medium was added. The dishes were illuminated with 666 nm light for $c_{e6}$ conjugates or 654 nm light for $c_{e6}$ at a power density of 48 mW $cm^{-2}$, measured with a power meter (Model 210, Coherent Inc., Palo Alto, CA, USA). An argon-pumped dye laser (Innova 100 and 599 Dye, Coherent) was focused through a ×10 microscope lens onto the end of a 1 mm diameter optical fibre which delivered light through an inverted ×4 microscope lens (No 774317, Olympus, Tokyo, Japan) to give a 35 mm diameter spot for irradiation. Controls were as follows: no conjugate and no light remaining in incubator throughout, no conjugate and no light but plates wrapped in foil for duration of the irradiation time out of the incubator, conjugate given and no light wrapped in foil, no conjugate and irradiated. At the completion of irradiation the cells were given fresh medium containing FCS and returned to the incubator for 24 h. The cells were then washed with medium and any detached cells aspirated off. The remaining cells were detached with trypsin/EDTA (0.5 ml) and an aliquot counted for viable cells using the trypan blue exclusion assay and a haemocytometer. The cell suspension was diluted with medium and plated in P60 dishes containing 4 ml of medium at densities of 50, 100, 150 and 200 cells per plate. When the colonies had formed (9 days later) the cells were fixed with 0.2% formalin (vol/vol) in MeOH and stained with crystal violet. The number of colonies, which contained 50 or more cells, was then counted. The survival fraction was calculated by multiplying the fraction of viable cells at the counting stage compared to controls (sensitizer, dark, out of incubator), together with the fraction of colonies formed by treated cells compared to controls.

### RESULTS

### Absorbance spectroscopy

The absorption spectra of the cationic and anionic PICs together with unconjugated $c_{e6}$ are shown in Figure 2. The conjugates have a distinct absorbance at 280 nm due to the protein in the IgG, the intensity of the Soret band is somewhat reduced, and the long wavelength Q band is red shifted to 666 nm as opposed to 654 nm. It can be calculated assuming extinction coefficients of $1.5 \times 10^5$ $lmol^{-1}$ $cm^{-1}$ for $c_{e6}$ at the Soret band and $2.4 \times 10^5$ $lmol^{-1}$ $cm^{-1}$ for IgG at 280 nm, that the anionic 17.1A-pl-$c_{e6}$-succ had 8–9 $c_{e6}$ molecules attached to each IgG (two polylysine chains per IgG), while the cationic 17.1A-pl-$c_{e6}$ had a lower loading of 4–5 $c_{e6}$ molecules per IgG (one polylysine chain per IgG).

© 2000 Cancer Research Campaign

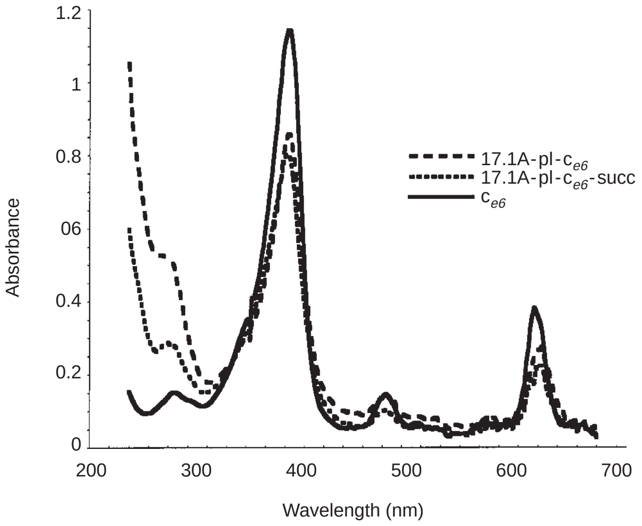

**Figure 2** Absorption spectra. Conjugates and free $c_{e6}$ were dissolved in 0.1 M NaOH/1% SDS at a concentration of 7 µM $c_{e6}$ equivalent

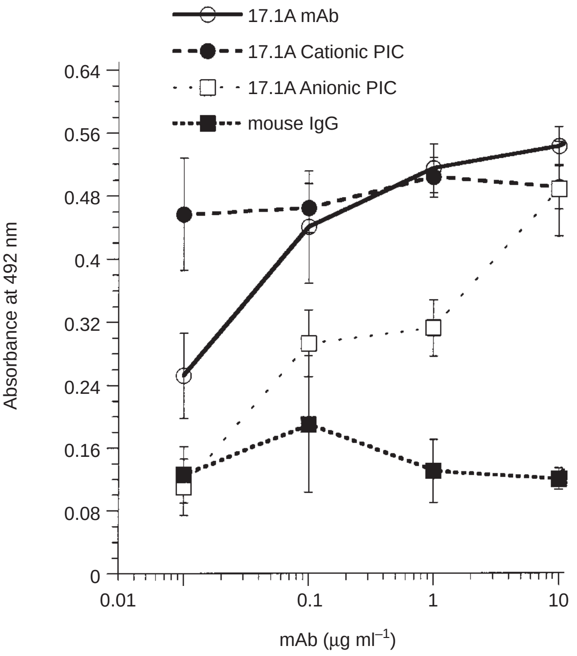

**Figure 3** ELISA assays on fixed cells. Fixed cells were incubated with a dilution of mAb or PIC, then treated with HRP-conjugated rabbit anti-mouse F(ab′)2 fragment and colour developed with o-phenylenediamine and $H_2O_2$. Each point is the mean of six wells and bars are the s.e.m.

### ELISA

Results of ELISA binding assays using fixed cells and assaying binding of 17.1A mAb, 17.1A-pl-$c_{e6}$, 17.1A-pl-$c_{e6}$-succ and non-specific mouse IgG are shown in Figure 3. The unmodified mAb exhibited a typical binding curve with reduction in binding over the concentration range 10–0.1 µg ml$^{-1}$ protein. The anionic 17.1A-pl-$c_{e6}$-succ showed a somewhat reduced affinity, while the cationic 17.1A-pl-$c_{e6}$ showed a slightly higher affinity compared to native 17.1A. As expected, non-specific mouse IgG showed no binding.

### Two-colour direct/indirect immunofluorescence microscopy

Two-colour direct/indirect immunofluorescence microscopy was carried out by treating fixed cells with a PIC, which binds to membrane antigens, and then adding a FITC-conjugated goat anti-mouse second antibody that recognizes the murine IgG of the first mAb. Fluorescent images were obtained with a dual filter system capable of isolating green fluorescence (510 nm) emitted by FITC, from red fluorescence (670 nm) emitted by $c_{e6}$. Non-matching filter/fluorophore combinations, i.e. green emission filter and $c_{e6}$ alone, and red emission filter and FITC alone gave negative images (data not shown). Images with similar appearance were obtained with both direct $c_{e6}$ and indirect FITC fluorescent emissions from 17.1A-pl-$c_{e6}$ bound to fixed HT29 cells (Figure 4 A, B). In a similar fashion images obtained from 17.1A-pl-$c_{e6}$-succ with both $c_{e6}$ and FITC emissions were strikingly similar (Figure 4 C, D).

### Cellular binding and uptake

The uptake of $c_{e6}$ per mg cell protein from the 17.1A-pl-$c_{e6}$ and 17.1A-pl-$c_{e6}$-succ in a range of concentrations of PICs (measured as $c_{e6}$ equivalent in the medium) is shown in Figure 5. The uptake of $c_{e6}$ equivalent from the cationic PIC is up to 4 times higher than that obtained from the anionic PIC after 6 h incubation, and both show linear relationships with increasing concentration. This linear increase is consistent with internalization of the conjugates. A concentration of 3 µM $c_{e6}$ equivalent is equivalent to approximately 300–500 nM 17.1A, which is much higher than the binding constant of the typical mAb. Thus if there was no internalization some saturation of uptake should have been observed. The cellular uptake of $c_{e6}$ obtained with the 17.1A PICs were compared to that obtained with non-specific rabbit IgG PICs, and free $c_{e6}$ (Table 1) under the same incubation conditions (1 µM $c_{e6}$ equivalent, 6 h incubation at 37°C in serum containing medium). Free $c_{e6}$ gave the lowest uptake followed by rabbit IgG-pl-$c_{e6}$-succ and 17.1A-pl-$c_{e6}$-succ. The cationic rabbit IgG-pl-$c_{e6}$ gave roughly twice the uptake of its anionic counterpart. The 17.1A conjugates gave the highest uptakes among conjugates bearing the same charge, and the cationic 17.1A-pl-$c_{e6}$ gave 3 times the uptake of the anionic 17.1A-pl-$c_{e6}$-succ.

**Table 1** Comparison of uptake after incubation at 37°C and 4°C

| | Uptake at 37°C | Uptake at 4°C | Ratio |
|---|---|---|---|
| 17.1A-pl-ce6 | 2.4 ± 0.17 | 1.4 ± 0.053 | 1.71 |
| 17.1A-pl-ce6-succ | 0.73 ± 0.037 | 0.23 ± 0.006 | 3.17 |
| Rabbit IgG-pl-ce6 | 1.1 ± 0.064 | 0.65 ± 0.035 | 1.53 |
| Rabbit IgG-pl-ce6-succ | 0.32 ± 0.022 | 0.071 ± 0.003 | 4.5 |
| ce6 | 0.063 ± 0.02 | 0.025 ± 0.002 | 2.47 |

Uptake was determined after incubation with 1 µM $c_{e6}$ equivalent concentration for 6 h. Concentrations are expressed as nmol $c_{e6}$ equivalent/mg cell protein. Each value is the mean of values from two separate experiments each containing three wells ± s.e.m.

© 2000 Cancer Research Campaign

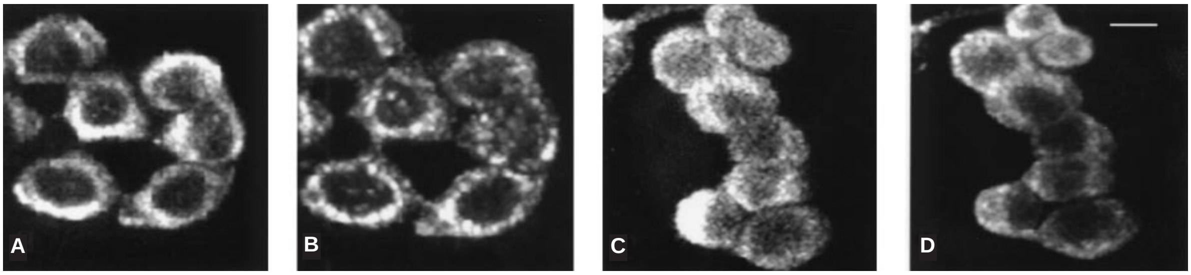

**Figure 4** Two-colour direct/indirect immunofluorescence microscopy with fixed HT29 cells incubated with PIC at 2 μg ml$^{-1}$ protein concentration, followed by FITC-conjugated goat anti-mouse second antibody and imaged with a filter set which isolated green from red fluorescence. Bar represents 20 μm. (**A, B**) 17.1A-pl-$c_{e6}$, (**A**) $c_{e6}$ fluorescence, (**B**) FITC fluorescence. (**C, D**) 17.1A-pl-$c_{e6}$-succ, (**C**) $c_{e6}$ fluorescence, (**D**) FITC fluorescence

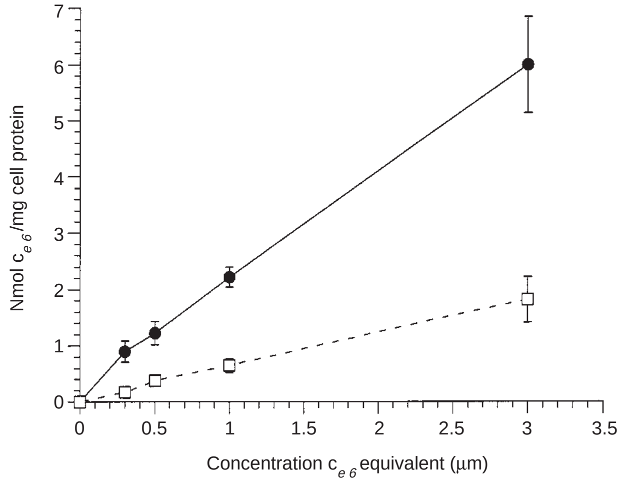

**Figure 5** Concentration dependence of cellular uptake of $c_{e6}$ from cationic 17.1A-pl-$c_{e6}$ and anionic 17.1A-pl-$c_{e6}$-succ. Cells were incubated for 6 h at 37°C for each PIC and cellular fluorescence was measured after extraction into 0.1 M NaOH/1% SDS and expressed in nmol $c_{e6}$ per mg cell protein. Each point is the mean of values from two separate experiments each containing three wells and bars are s.e.m.

**Table 2** Selectivity of conjugates for HT29 cells compared to OVCAR-5 cells

| Conjugate | HT29 cells | OVCAR-5 cells | Selectivity for HT29 |
|---|---|---|---|
| 17.1A-pl-$c_{e6}$ | 2.45 ± 0.17 | 1.3 ± 0.06 | 1.9 : 1 ($P$ < 0.01) |
| 17.1A-pl-$c_{e6}$-succ | 0.74 ± 0.04 | 0.34 ± 0.02 | 2.2 : 1 ($P$ < 0.01) |
| Rabbit IgG-pl-$c_{e6}$- | 1.0 ± 0.06 | 0.9 ± 0.01 | 1.1 : 1 (n.s.) |
| Rabbit IgG-pl-$c_{e6}$-succ | 0.29 ± 0.01 | 0.34 ± 0.02 | 0.9 : 1 (n.s.) |

Uptake was determined after incubation at 1 μM $c_{e6}$ equivalent concentration for 6 h at 37°C using non-specific rabbit IgG anionic and cationic PICs and 17.1A cationic and anionic PICs. Cellular fluorescence was measured after extraction into 0.1 M NaOH/1% SDS and expressed in nmol $c_{e6}$ equivalent/mg cell protein. Each value is the mean of values from two separate experiments each containing three wells ± s.e.m. Significance was assessed by two-tailed unpaired Student's *t*-test.

### Effect of incubation temperature on uptake

In order to gain additional information on the degree to which the PIC which is bound to the cells is internalized, the uptake after incubation at 37°C was compared to that found after incubation at 4°C. The concentration was 1 μM $c_{e6}$ equivalent and the incubation time was 6 h. The results are given in Table 1. It can be seen that the cationic species have about 65% of the uptake remaining at 4°C, while the anionic conjugates have only 30% remaining. However, the absolute amount of the conjugate internalized (difference between uptakes at 37°C and 4°C is greater for the cationic species than the anionic species. Note that the uptake of free $c_{e6}$ is also significantly greater at 37°C than 4°C.

### Comparison of uptake with non-target cell line

OVCAR-5 human ovarian carcinoma cell line was used as a non-target control compared to HT29 cells. Indirect immunofluorescence using 17.1A mAb and FITC-goat anti-mouse IgG on fixed OVCAR-5 cells gave a negative fluorescence image (data not shown) indicating that OVCAR-5 cells expressed very low levels of EpCAM, but the two-colour direct/indirect immunofluorescence procedure with both the cationic and anionic 17.1A PICs gave weak images for both $c_{e6}$ and FITC fluorescence (data not shown) indicating a small amount of non-specific binding of the PICs to OVCAR-5 cells.

The uptake of $c_{e6}$ by live OVCAR-5 and HT29 cells from the cationic and anionic 17.1A PICs and from cationic and anionic non-specific rabbit IgG PICs was compared and the results are shown in Table 2. Almost no difference was found between target and non-target cell line using either the cationic and anionic non-specific rabbit IgG PICs as expected, whereas the uptake from both the cationic and anionic 17.1A PIC showed twofold selectivity for the target HT29 cells compared to the non-target OVCAR-5 cells.

### Competition of uptake by unmodified proteins

In order to further understand to what extent the observed uptake was due to antigen binding and what extent to non-specific charge interaction, uptake of $c_{e6}$ from cationic and anionic 17.1A PICs by HT29 cells was compared with and without saturation of the antigens by preincubation with unmodified 17.1A (Figure 6). The uptake of $c_{e6}$ from the cationic 17.1A-pl-$c_{e6}$ was reduced to 30% of the control level by preincubation with 17.1A, while that of the anionic 17.1A-pl-$c_{e6}$-succ was reduced to 10% of the control level. That this reduction was due to blocking of the antigenic binding sites by 17.1A is confirmed by the observation that preincubation with rabbit IgG had no effect on subsequent binding of 17.1A PICs. As expected the uptake of the cationic PIC had a greater contribution from non-antigen mediated charge interactions than the anionic PIC.

 *© 2000 Cancer Research Campaign*

### Phototoxicity

Cells were illuminated after 6 h incubation with 1 μM $c_{e6}$ equivalent of all the compounds. The phototoxicity was measured by using a colony forming assay that combines short-term and long-term damage to the cells. The 17.1A cationic and anionic PICs (with HT29 target cells) showed fluence-dependent phototoxicity with greater than 99.9% killing after 10 J $cm^{-2}$ 666 nm red light (Figure 7A). To compare the phototoxicities of different pairings of cells and PICs we carried out experiments at a constant fluence of 3 J $cm^{-2}$ and the results are shown in Figure 7B. Under these conditions 10% of HT29 cells survived after treatment with 17.1A-pl-$c_{e6}$ and 27% survived after PIT with 17.1A-pl-$c_{e6}$-succ. Comparing these results with the killing produced by the non-specific rabbit IgG PICs of the same overall charges the difference is seen to be significant; four times the number of surviving of cells with the rabbit IgG cationic PIC compared to the 17.1A cationic PIC, and three times more cells surviving after PIT with the anionic rabbit IgG PIC compared to the 17.1A anionic PIC. The free $c_{e6}$ had a very low phototoxicity compared to both the 17.1A PICs. Cell type selectivity of the 17.1A PICs was shown by using OVCAR-5 cells as a non target cell line; the survival was 3–4 times higher than the target HT29 cell line gave. Interestingly OVCAR-5 cells were killed by PIT with the 17.1A cationic and anionic conjugates to almost exactly the same extent as the non-specific rabbit IgG PICs killed HT29 cells as shown in Figure 7B.

### DISCUSSION

Additional therapies to target colorectal cancer metastases in the liver are urgently needed (Van Cutsem, 1996). Existing treatments include surgery (Steele and Ravikumar, 1989), chemoembolization (Sanz-Altamira et al, 1997), regional chemotherapy (McMurrick and Nelson, 1997) and cryotherapy (Yeh et al, 1997). Among investigative treatments some workers are exploring the role of PDT (Van Hillegersberg et al, 1992*a*). A drawback of PDT in the liver is the high accumulation of most free PS in normal liver (Van Hillegersberg et al, 1992*b*). The preparation of PICs that could target tumour-associated antigens on the colorectal cancer metastasis as opposed to normal liver tissue may increase the specificity of PDT for liver metastases. In addition the disseminated intraperitoneal spread of colorectal cancer may also be treated by PDT (Veenhuizen et al, 1996), but again additional selectivity for the tumour is necessary to prevent unwanted

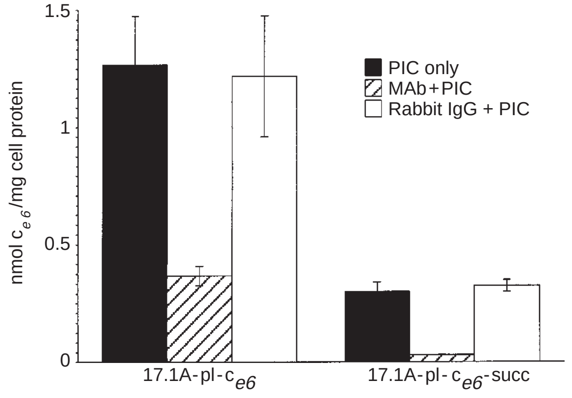

**Figure 6** Inhibition of uptake by preincubation of cells with unmodified Mab. Cells were incubated for 2 h at 37°C with the 17.1A cationic or anionic PICs at the concentration of 1 μM $c_{e6}$ equivalent that contains 7.5 μg 17.1A mAb (PIC only). Other dishes of cells were incubated for the first hour with the unmodified 17.1A mAb or unmodified rabbit IgG at the amount of 37.5 μg (fivefold excess over the protein contained in the PICs) and for a further hour with 17.1A cationic or anionic PICs (mAb+PIC) or (rabbit IgG + PIC). Cellular fluorescence was measured after extraction into 0.1 M NaOH/1% SDS and expressed in nmol $c_{e6}$/mg cell protein. Each point is the mean of values from two separate experiments each containing three wells and bars are s.e.m.

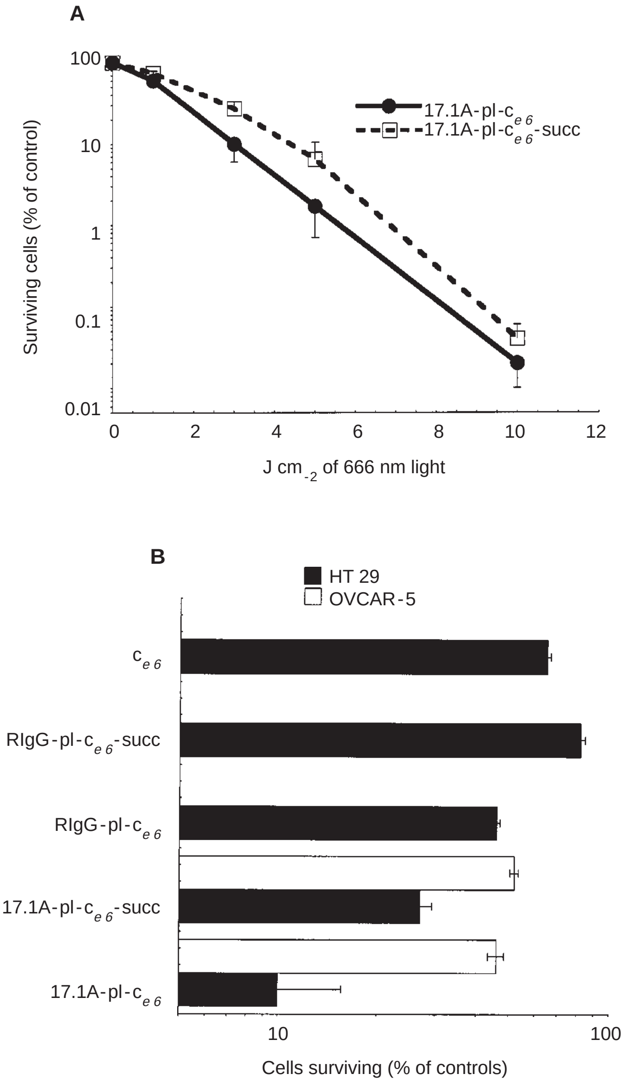

**Figure 7** Phototoxicity of conjugates. (**A**) Fluence response survival curve for HT29 cells treated with 17.1A-pl-$c_{e6}$ and 17.1A-pl-$c_{e6}$-succ. (**B**) Comparison of survival of HT29 cells and OVCAR-5 cells treated with specific and non-specific PICs at a fluence of 3 J $cm^{-2}$. Cells were incubated for 6 h at 37°C with 1 μM $c_{e6}$ equivalent of: free $c_{e6}$, non-specific cationic rabbit IgG-pl-$c_{e6}$ and anionic rabbit IgG-pl-$c_{e6}$-succ, cationic 17.1A-pl-$c_{e6}$ and anionic 17.1A-pl-$c_{e6}$-succ. Cells were illuminated in a P35 dishes using 666 nm light (conjugates) and 654 nm ($c_{e6}$). Results were measured by a colony forming ability after 9 days and are expressed in % cells surviving compared to dark controls treated with PS or conjugate. For each point three separate plates of cells were given PIT and each plate further plated for colonies in four dilutions. Values are the means of survival fractions obtained and bars are s.e.m.

© 2000 Cancer Research Campaign

**Table 3** Relative phototoxicity per unit cellular uptake of $c_{e6}$ delivered by conjugates and free $c_{e6}$

| Conjugate | HT29 cells | OVCAR-5 cells |
|---|---|---|
| 17.1A-pl-$c_{e6}$ | 4.17 ± 0.56 | 2.17 ± 0.06 |
| 17.1A-pl-$c_{e6}$-succ | 5.07 ± 0.11 | 4.79 ± 0.07 |
| Rabbit IgG-pl-$c_{e6}$ | 2.17 ± 0.07 | |
| Rabbit IgG-pl-$c_{e6}$-succ | 3.59 ± 0.07 | |
| $c_{e6}$ | 24.5 ± 1.31 | |

The phototoxicity (1/survival fraction) after incubation at 37°C for 6 h at 1 μM $c_{e6}$ equivalent concentration and 3 J cm$^{-2}$ 666 or 654 nm light, was divided by the cellular uptake in nmol $c_{e6}$ equivalent per mg cell protein. Errors are the s.e.m. of the ratio of the means calculated in quadrature.

damage to intestines and other intraperitoneal organs (Veenhuizen et al, 1997). There are many reports in the literature which confirm the selectivity of the 17.1A murine mAb toward gastrointestinal tumours and in particular to colorectal cancer (Martin et al, 1986; Pierce et al, 1990; Buchsbaum et al, 1993*b*; Meredith et al, 1995). In order for PIT to be effective each PIC molecule must deliver as much PS as possible to the tumour, without sacrificing unduly the specificity and affinity of the PIC for its antigen. One way of accomplishing this is to use a polymeric linker to attach several PS molecules in a site-specific manner to the mAb, and this linker may bear positive, negative or neutral charge (Hamblin et al, 1996). Variation in the overall charge may affect the binding of the PIC to its target antigen, its intracellular location and its phototoxicity. A previous publication reported the effect of charge on the selectivity, uptake and phototoxicity of OC125 F(ab′)$_2$ PICs constructed in a similar fashion to the present 17.1A PICs (Hamblin et al, 1996). Alterations in the charge borne by mAb conjugates can also lead to wide variations in the biodistribution and pharmacokinetics (Slinkin et al, 1993; Duska et al, 1997). The object of this study was to investigate the binding (specificity and affinity) of cationic and anionic 17.1A PICs to HT29 target human colorectal cancer cells, and their consequent phototoxicity.

The results from the ELISA and two-colour direct/indirect immunofluorescence studies on fixed cell, showed that the capacity of the two differently charged PICs to recognize the membrane antigen expressed on HT29 cells, compared reasonably with that of unmodified 17.1A. Since these cells were fixed, the subsequent endocytosis of bound PICs was not an issue. Although the affinity of the anionic PIC was somewhat reduced as measured by the ELISA it was still relatively high. The fluorescence microscopy showed that with fixed cells the 17.1A and the $c_{e6}$ delivered by the PIC had very similar localizations making it likely that the great majority of $c_{e6}$ delivered to the cells was covalently linked to the mAb.

When the PICs were administered to living cells considerable differences in the uptake of $c_{e6}$ between the opposite charged PICs were observed. The cationic PIC delivered an average of 3.5 times more $c_{e6}$ than the anionic PIC. This is a similar but smaller multiple to that found with cationic and anionic OC125 F(ab′)$_2$ PICs and their target OVCAR-5 cells in a previous study (Hamblin et al, 1996), where the enhanced $c_{e6}$ uptake from the cationic PIC was attributed to increased rates of internalization of polycationic mAbs compared to uncharged or polyanionic species. The relative $c_{e6}$ uptake of the cationic species relative to free $c_{e6}$ was 38:16:1 for 17.1A PIC, rabbit IgG PIC and $c_{e6}$ respectively, and for the anionic species was 11.5:5.5:1 respectively. It seems therefore that the antigen mAb recognition at least doubles the uptake compared to other conjugates of the same charge. The data comparing uptake at 37°C and 4°C show that the cationic conjugates bind much better than the anionic ones to the membrane at 4°C, and the additional internalized uptake at 37°C is only another 50%; while for the anionic conjugates the additional uptake at 37°C was 200–300% of that at 4°C. This suggests that a higher proportion of the anionic 17.1A PIC was internalized than for the cationic 17.1A PIC, although the overall uptake was less. Uptake of $c_{e6}$ from both the cationic and anionic 17.1A PICs was also approximately twofold higher by target HT29 colorectal cancer cells than by non-target OVCAR-5 ovarian cancer cells, while the non-specific rabbit IgG PICs showed no difference in $c_{e6}$ uptake between cell lines. However, since the EpCAM antigen is a common antigen overexpressed on cancer cells it is possible that OVCAR-5 cells also expressed this antigen, although the indirect immunofluorescence was negative. Additional evidence of the retention of antigen recognition in the PICs was provided by the experiments in which the antigen was saturated by preincubation with unmodified mAb. It should be noted that it is difficult to get any selectivity between two epithelial human cancer cell lines using unconjugated PS in vitro.

One of the critical issues to be considered in immunoconjugate therapy is the penetration of the immunoconjugate into the tumour tissue (Jain, 1990). It has been shown (Saga et al, 1995) that high affinity mAbs penetrate less well than those of lower affinity, and therefore a conjugation process which reduces the affinity of the mAb for target antigen expressing cells, may be quite acceptable.

The question then arises, to what extent do the values for $c_{e6}$ uptake delivered by the various conjugates correlate with the phototoxicity? Under the conditions where the uptake of $c_{e6}$ from the cationic conjugates relative to unconjugated $c_{e6}$ was 38:16:1 for 17.1A and rabbit IgG, the relative phototoxicity (1/survival fraction) compared to unconjugated $c_{e6}$ with 3 J cm$^{-2}$ 666 nm light was 6.5:1.4:1. Similarly for the anionic series where the relative uptakes were 11.5:5.5:1, the relative phototoxicities were 2.4:0.8:1. To determine which of these conjugates is inherently the most effective photosensitizer, we can divide the relative phototoxicity by the uptake in nmol $c_{e6}$ equivalent per mg cell protein and the resulting numbers are shown in Table 2. Free $c_{e6}$ would appear to have more phototoxic potential than any of the conjugates per unit $c_{e6}$ uptake, but since the uptake is exceptionally low this is not of much relevance. This is in agreement with other reports (Bachor et al, 1991) that free $c_{e6}$ has such low cellular uptake that it is difficult to get any phototoxicity in vitro. The remaining conjugates have roughly similar values but the values obtained when mAb conjugates interact with target cells are consistently higher than non-matching pairs. These results can be compared with those obtained (Hamblin et al, 1996) with polycationic and polyanionic OC125F(ab′)$_2$ conjugates prepared in a similar manner and directed towards target OVCAR-5 cells. In this previous study the charge effect was found to be of larger magnitude but in the same direction as the present study, with the polycationic PIC having 6 times the uptake and 10 times the phototoxicity of the polyanionic PIC. This difference in magnitude between charge effects may be attributed to differences in the magnitude of the negative charge expressed on the outside of the cells (Bischoff et al, 1981), or to differences in the extent to which polycations stimulate endocytosis between cell lines (Duncan et al, 1979). It is accepted that imparting cationic charge to a protein

© 2000 Cancer Research Campaign

(Shen and Ryser, 1978), receptor ligand (Cotten et al, 1990), mAb (Pardridge et al, 1994) or immunoconjugate (Hamblin et al, 1996) increases the absolute uptake and the degree to which it is internalized in vitro. It is thought that the mechanism of increased uptake is due to non-clathrin-coated pit-mediated endocytosis leading to endosomal processing (Hansen et al, 1993), and to accumulation in lysosomes where proteolysis may take place. In vivo, however, polycationic moieties have high and fast uptake in the liver and kidney when administered i.v. (Clegg et al, 1990), which suggest that polycationic PICs would be better suited to intracavitary administration (Hamblin et al, 1996). This hypothesis was confirmed with a series of biodistribution experiments conducted with OC125F(ab′)$_2$ PICs prepared in a similar manner to the present PICs and injected i.p. in nude mice bearing i.p. OVCAR-5 tumours (Duska et al, 1997). However, in order to effectively target colorectal tumour cells growing in the liver where the PIC must be administered i.v., it is likely that a *polyanionic* PIC will outperform a *polycationic* species. This hypothesis will be tested in a forthcoming report.

In conclusion we have demonstrated advantages of conjugating $c_{e6}$ to mAb 17.1A by a site-specific synthetic route. The immunoreactivity is preserved, the PICs show selectivity to target colorectal cancer cells over non-target ovarian cancer cells, and the absolute uptake by tumour cells is very much higher for both charges than that given by the free $c_{e6}$. In vitro there is little difference in the amount of killing per molecule of $c_{e6}$ delivered by polycationic and polyanionic PICs, thus leaving the choice of charge borne by the PIC for in vivo PIT to be made on the basis of biodistribution and pharmacokinetic data. While these initial data on the preferential photodestruction of target cells are encouraging, there remain many questions which will only be answered by in vivo experiments. Will the PICs be able to penetrate solid tumours after i.v. administration? In addition to binding to tumour cells, will the PICs be taken up by cells of the monocyte/macrophage lineage which are especially prevalent in liver? Will the pharmacokinetics and biodistribution of the PICs be suitable for effective photodestruction of tumours in vivo? These questions will be addressed in forthcoming reports.

### ACKNOWLEDGEMENTS

This work was supported by NIH Grant number R01 AR 40352 and facilities by the ONR-MFEL program contract number N 00 014-94-1-0927.

We thank Dr K Tanabe for his generous gift of HT-29 cells, Centacor Inc. for the gift of 17.1A mAb, and MP Bamberg for technical assistance.

### REFERENCES

Bachor R, Shea CR, Gillies R and Hasan T (1991) Photosensitized destruction of human bladder carcinoma cells treated with chlorin e6-conjugated microspheres. *Proc Natl Acad Sci USA* **88**: 1580–1584

Barr H, Krasner N, Boulos PB, Chatlani P and Bown SG (1990) Photodynamic therapy for colorectal cancer: a quantitative pilot study. *Br J Surg* **77:** 93–96

Benotti P and Steele G (1992) Patterns of recurrent colorectal cancer and recovery surgery. *Cancer* **70**: 1409–1413

Bischoff P, Robert F and Donner M (1981) A comparative study of some growth characteristics and cell-surface properties of neoplastic cells. *Br J Cancer* **44**: 545–552

Buchsbaum DJ, Langmuir VK and Wessels BW (1993*a*) Experimental radioimmunotherapy. *Med Phys* **20**: 551–567

Buchsbaum DJ, Lawrence TS, Roberson PL, Heidorn DB, Ten Haken RK and Steplewski Z (1993*b*). Comparison of 131I- and 90Y-labeled monoclonal antibody 17-1A for treatment of human colon cancer xenografts. *Int J Radiat Oncol Biol Phys* **25**: 629–638

Clegg JA, Hudecz F, Mezo G, Pimm MV, Szekerke M and Baldwin RW (1990) Carrier design: biodistribution of branched polypeptides with a poly(L-lysine) backbone. *Bioconjug Chem* **1**: 425–430

Cotten M, Langle-Rouault F, Kirlappos H, Wagner E, Mechtler K, Zenke M, Beug H and Birnstiel ML (1990) Transferrin-polycation-mediated introduction of DNA into human leukemic cells: stimulation by agents that affect the survival of transfected DNA or modulate transferrin receptor levels. *Proc Natl Acad Sci USA* **87**: 4033–4037

Dougherty TJ, Gomer CJ, Henderson BW, Jori G, Kessel D, Korbelik M, Moan J and Peng Q (1998) Photodynamic therapy. *J Natl Cancer Inst* **90**: 889–905

Duncan R, Pratten MK and Lloyd JB (1979) Mechanism of polycation stimulation of pinocytosis. *Biochim Biophys Acta* **587**: 463–475

Duska LR, Hamblin MR, Bamberg MP and Hasan T (1997) Biodistribution of charged F(ab′)$_2$ photoimmunoconjugates in a xenograft model of ovarian cancer. *Br J Cancer* **75**: 837–844

Goff BA, Bamberg M and Hasan T (1991) Photoimmunotherapy of human ovarian carcinoma cells ex vivo. *Cancer Res* **51**: 4762–4767

Goff BA, Hermanto U, Rumbaugh J, Blake J, Bamberg M and Hasan T (1994) Photoimmunotherapy and biodistribution with an OC125-chlorin immunoconjugate in an in vivo murine ovarian cancer model. *Br J Cancer* **70**: 474–480

Goff BA, Blake J, Bamberg MP and Hasan T (1996) Treatment of ovarian cancer with photodynamic therapy and immunoconjugates in a murine ovarian cancer model. *Br J Cancer* **74**: 1194–1198

Hamblin MR and Newman EL (1994) On the mechanism of the tumour-localising effect in photodynamic therapy. *J Photochem Photobiol B* **23**: 3–8

Hamblin MR, Miller JL and Hasan T (1996) The effect of charge on the interaction of site-specific photoimmunoconjugates with human ovarian cancer cells. *Cancer Res* **56**: 5205–5210

Hansen SH, Sandvig K and van Deurs B (1993) Molecules internalized by clathrin-independent endocytosis are delivered to endosomes containing transferrin receptors. *J Cell Biol* **123**: 89–97

Hasan T (1992) Photosensitizer delivery mediated by macromolecular carrier systems. In *Photodynamic Therapy: Basic Principles and Clinical Applications*, Henderson B and Dougherty T (eds), pp. 187–200. Marcel Dekker: London

Hasan T and Parrish JA (1996) Photodynamic therapy of cancer. In: *Cancer Medicine*, Holland JF, Frei EI, Bast RCJ, Kufe DW, Morton DL and Weichselbaum RR (eds), pp. 739–751. Williams & Wilkins: Baltimore

Henderson BW and Dougherty TJ (1992) How does photodynamic therapy work? *Photochem Photobiol* **55**: 145–157

Houston LL (1993) Targeted delivery of toxins and enzymes by antibodies and growth factors. *Curr Opin Biotechnol* **4**: 739–744

Jain RK (1990) Physiological barriers to delivery of monoclonal antibodies and other macromolecules in tumours. *Cancer Res* **50**: 814s–819s

Johnson DA, Briggs SL, Gutowski MC and Barton R (1995) Anti-tumor activity of CC49-doxorubicin immunoconjugates. *Anticancer Res* **15**: 1387–1393

Kornguth SE, Kalinke T, Robins HI, Cohen JD and Turski P (1989) Preferential binding of radiolabeled poly-L-lysines to C6 and U87 MG glioblastomas compared with endothelial cells in vitro. *Cancer Res* **49**: 6390–6395

Larson E, Howlett B and Jagendorf A (1986) Artificial reductant enhancement of the Lowry method for protein determination. *Anal Biochem* **155**: 243–248

Litvinov SV, Velders MP, Bakker HA, Fleuren GJ and Warnaar SO (1994) Ep-CAM: a human epithelial antigen is a homophilic cell–cell adhesion molecule. *J Cell Biol* **125**: 437–446

McMurrick PJ and Nelson H (1997) Liver-directed therapies for gastrointestinal malignancies. *Curr Opin Oncol* **9**: 367–372

Martin EW, Jr, Tuttle SE, Rousseau M, Mojzisik CM, O'Dwyer PJ, Hinkle GH, Miller EA, Goodwin RA, Oredipe OA, Barth RF & et al. (1986) Radioimmunoguided surgery: intraoperative use of monoclonal antibody 17-1A in colorectal cancer. *Hybridoma* **5**: S97–S108

Meredith RF, Khazaeli MB, Plott WE, Spencer SA, Wheeler RH, Brady LW, Woo DV and LoBuglio AF (1995) Initial clinical evaluation of iodine-125-labeled chimeric 17-1A for metastatic colon cancer. *J Nucl Med* **36**: 2229–2233

Mew D, Wat CK, Towers GH and Levy JG (1983) Photoimmunotherapy: treatment of animal tumors with tumor-specific monoclonal antibody-hematoporphyrin conjugates. *J Immunol* **130**: 1473–1477

Pardridge WM, Bickel U, Buciak J, Yang J and Diagne A (1994) Enhanced endocytosis and anti-human immunodeficiency virus type 1 activity of anti-rev antibodies after cationization. *J Infect Dis* **169**: 55–61

Pierce DL, Heindel ND, Schray KJ, Jetter MM, Emrich JG and Woo DV (1990) Misonidazole conjugates of the colorectal tumor associated monoclonal antibody 17-1A. *Bioconjug Chem* **1**: 314–318

Pogrebniak HW, Matthews W, Black C, Russo A, Mitchell JB, Smith P, Roth JA and Pass HI (1993) Targetted phototherapy with sensitizer-monoclonal antibody conjugate and light. *Surg Oncol* **2**: 31–42

Riethmuller G, Schneider-Gadicke E, Schlimok G, Schmiegel W, Raab R, Hoffken K, Gruber R, Pichlmaier H, Hirche H, Pichlmayr R & et al. (1994) Randomised trial of monoclonal antibody for adjuvant therapy of resected Dukes' C colorectal carcinoma. German Cancer Aid 17-1A Study Group. *Lancet* **343**: 1177–1183.

Saga T, Neumann RD, Heya T, Sato J, Kinuya S, Le N, Paik CH and Weinstein JN (1995) Targeting cancer micrometastases with monoclonal antibodies: a binding-site barrier. *Proc Natl Acad Sci USA* **92**: 8999–9003

Sanz-Altamira PM, Spence LD, Huberman MS, Posner MR, Steele G, Jr, Perry LJ and Stuart KE (1997) Selective chemoembolization in the management of hepatic metastases in refractory colorectal carcinoma: a phase II trial. *Dis Colon Rectum* **40**: 770–775

Schmidt S, Wagner U, Oehr P and Krebs D (1992) Clinical use of photodynamic therapy in gynecologic tumor patients–antibody-targeted photodynamic laser therapy as a new oncologic treatment procedure. *Zentralbl Gynakol* **114**: 307–311

Shen WC and Ryser HJ (1978) Conjugation of poly-L-lysine to albumin and horseradish peroxidase: a novel method of enhancing the cellular uptake of proteins. *Proc Natl Acad Sci USA* **75**: 1872–1876

Slinkin MA, Curtet C, Faivre-Chauvet A, Sai-Maurel C, Gestin JF, Torchilin VP and Chatal JF (1993) Biodistribution of anti-CEA F(ab′)2 fragments conjugated with chelating polymers: influence of conjugate electron charge on tumor uptake and blood clearance. *Nucl Med Biol* **20**: 443–452

Speiser PP (1991) Nanoparticles and liposomes: a state of the art. *Methods Find Exp Clin Pharmacol* **13**: 337–342

Steele G, Jr and Ravikumar TS (1989) Resection of hepatic metastases from colorectal cancer. Biologic perspective. *Ann Surg* **210**: 127–138

Van Cutsem E (1996) A glimpse of the future: new directions in the treatment of colorectal cancer. *Eur J Cancer* 32A*:* S23

Van Hillegersberg R, Marijnissen JP, Kort WJ, Zondervan PE, Terpstra OT and Star WM (1992*a*) Interstitial photodynamic therapy in a rat liver metastasis model. *Br J Cancer* **66**: 1005–1014

Van Hillegersberg R, Van den Berg JW, Kort WJ, Terpstra OT and Wilson JH (1992*b*) Selective accumulation of endogenously produced porphyrins in a liver metastasis model in rats. *Gastroenterology* **103**: 647–651

Veenhuizen RB, Marijnissen JP, Kenemans P, Ruevekamp-Helmers MC, Mannetje LW, Helmerhorst TJ and Stewart FA (1996) Intraperitoneal photodynamic therapy of the rat CC531 adenocarcinoma. *Br J Cancer* **73**: 1387–1392

Veenhuizen RB, Ruevekamp MC, Oppelaar H, Ransdorp B, van de Vijver M, Helmerhorst TJ, Kenemans P and Stewart FA (1997) Intraperitoneal photodynamic therapy: comparison of red and green light distribution and toxicity. *Photochem Photobiol* **66**: 389–395

Vrouenraets MB, Visser GW, Stewart FA, Stigter M, Oppelaar H, Postmus PE, Snow GB and van Dongen GA (1999) Development of meta-tetrahydroxyphenylchlorin–monoclonal antibody conjugates for photoimmunotherapy. *Cancer Res* **59**: 1505–1513

Yeh KA, Fortunato L, Hoffman JP and Eisenberg BL (1997) Cryosurgical ablation of hepatic metastases from colorectal carcinomas. *Am Surg* **63**: 63–68

*© 2000 Cancer Research Campaign*

*© 2000 Cancer Research Campaign*
